# The combined effect of *Pdx1* overexpression and Shh manipulation on the function of insulin‐producing cells derived from adipose‐tissue stem cells

**DOI:** 10.1002/2211-5463.12378

**Published:** 2018-02-05

**Authors:** Mahmoud Hashemi Tabar, Mohammad Reza Tabandeh, Eskandar Moghimipour, Dian Dayer, Ata A. Ghadiri, Elham Allah Bakhshi, Mahmoud Orazizadeh, Mohammad Ali Ghafari

**Affiliations:** ^1^ Cellular and Molecular Research center Ahvaz Jundishapur University of Medical Sciences Iran; ^2^ Department of Anatomy Faculty of Medicine Ahvaz Jundishapur University of Medical Sciences Iran; ^3^ Department of Biochemistry and Molecular Biology Faculty of Veterinary Medicine Shahid Chamran University of Ahvaz Iran; ^4^ Stem Cells and Transgenic Technology Research Center Shahid Chamran University of Ahvaz Iran; ^5^ Department of pharmaceutics Faculty of Pharmacy Ahvaz Jundishapur University of Medical Sciences Iran; ^6^ Department of Immunology Faculty of Medicine Ahvaz Jundishapur University of Medical Sciences Iran; ^7^ Department of Biochemistry Faculty of Medicine Ahvaz Jundishapur University of Medical Sciences Iran

**Keywords:** adipose tissue‐derived mesenchymal stem cells, insulin‐producing cells, *Pdx1*, sonic hedgehog pathway

## Abstract

Pancreatic and duodenal homeobox 1 (Pdx1) and Sonic hedgehog (Shh) are the key regulators of beta‐cell function. *In vitro* experiments have shown that there is significant cooperation between Pdx1 and Shh with regard to the production and maintenance of insulin‐producing cells (IPCs). In this study, the combined effect of *Pdx1* overexpression and Shh manipulation on the function of adipose tissue‐derived IPCs was determined. A eukaryotic expression vector (*Pdx1‐* pCDNA3.1(+)) was constructed and transfected into a Chinese hamster ovary (CHO) cell line. Adipose tissue‐derived mesenchymal stem cells (ADMSCs) obtained from rats were assigned to two groups [control (C) and manipulated (M)] and differentiated into IPCs. Manipulated cells were treated with a mixture of FGF‐β and cyclopamine and recombinant Shh protein at days 3 and 11, respectively, and transfected with *Pdx1‐* pCDNA3.1(+) at day 10. The expression of multiple genes related to function of beta cells was analyzed using real‐time PCR. The functionality of IPCs *in vitro* was analyzed through dithizone (DTZ) staining and ELISA. IPCs were injected into the tail vein of diabetic rats, and blood glucose and insulin concentrations were measured. CHO cells transfected with *Pdx1‐* pCDNA3.1(+) showed a significantly higher expression of *Pdx1* compared with nontransfected cells. Manipulated IPCs exhibited a significantly higher expression of *MafA, Nkx2.2, Nkx6.1, Ngn3, insulin*, and *Isl1* and a higher insulin secretion in response to glucose challenge in relation to control cells. Rats that received manipulated IPCs exhibited a higher ability to normalize blood glucose and insulin secretion when compared to controls. Our protocol might be used for more efficient cell therapy of patients with diabetes in the future.

**Abbreviations**
ADMSCs: adipose tissue‐derived mesenchymal stem cells
CDs: clusters of differentiation
CHO: Chinese hamster ovary
DAB: diaminobenzidine
DMEM: Dulbecco's modified Eagle's medium
DTZ: dithizone
IPCs: insulin‐producing cells
LB: lysogeny broth
NA: nicotinic acid
Pdx1: pancreatic and duodenal homeobox 1
Pen/Strep: penicillin/streptomycin
Shh: sonic hedgehog
T1DM: type 1 diabetes mellitus

Among the existing treatment methods for type 1 diabetes mellitus (T1DM), stem cell‐based therapy is a practical approach with permanent results 1. Several protocols have been proposed for the generation of functional insulin‐producing cells (IPCs). Nonetheless, the successful permanent transplantation of the generated cells to diabetic animal models for diabetes treatment requires the optimization of differentiation protocols 1. Adipose tissue‐derived mesenchymal stem cells (ADMSCs) present several advantages, including simple noninvasive access, a high reproduction capacity, a high differentiation potential, and the lack of immune system rejection or tumorigenesis 2. Some studies have shown a successful differentiation of ADMSCs toward functional IPCs 3. One desirable approach for optimizing diabetes cell‐based therapy is to modify the gene expression algorithm 4. Pancreatic and duodenal homeobox 1 (*Pdx1*) is a transcriptional activator of insulin promoter 5. In general, a high concentration of glucose induces the coordinated elevation of *Pdx1*, MafA, and NeuroD mRNA expression and ultimately results in the activation of insulin gene expression 6. *Pdx1* is viewed as an essential factor for pancreatic development and β‐cell maturation 7. Reduced *Pdx1* activity promotes the inhibition of insulin‐like growth factors and apoptotic β‐cell death 7. In humans, normal pancreas morphogenesis depends on early sonic hedgehog (Shh) suppression 8. Some studies have reported the reactivation of the Shh pathway during the late maturation of pancreatic β cells 9. Given the significant effect of Shh alteration on *Pdx1* gene expression and subsequent pancreatic β‐cell functionality 10, 11, a novel protocol is proposed in this study for analyzing the combined effects of *Pdx1* overexpression and Shh manipulation on the differentiation of ADMSCs toward IPCs.

## Materials and methods

### Isolation of rat tissues

Normal Sprague‐Dawley male rats aged 2–3 months and weighing 180–200 g were selected for the experiment. All the animals were kept in accordance with the Guide for the Care and Use of Laboratory Animals by the National Academy of Sciences (National Institutes of Health Publication No. 86‐23) and Ahvaz Jundishapur University of Medical Sciences (AJUMS.REC.1393.100). The rats were euthanized using a mixture of 100 mg·kg^−1^ ketamine and 10 mg·kg^−1^ xylazine. The pancreatic tissue and adipose tissue from the splanchnic region were isolated and washed three times with sterile PBS (Gibco, Waltham, MA, USA) containing 3% penicillin/streptomycin (Pen/Strep; Gibco).

### Construction of *Pdx1‐*pCDNA3.1(+)

Total RNA was isolated from the rats’ pancreas using RNX™ reagent as per the manufacturer's recommendations (SinaClon, Tehran, Iran). The RNA concentration was determined at the wavelength of 260 nm using a NanoDrop spectrophotometer (NanoDrop 2000™; Thermo Fisher Scientific, Waltham, MA, USA). The unimpaired RNA was confirmed by OD 260/280 nm between 1.8 and 2. The reverse transcription reaction was performed using CycleScript RT PreMix cDNA synthesis kit (Bioneer Corporation, Daejeon, South Korea) in the total amount of 20 μL as recommended by the manufacturer. PCR was carried out in the total volume of 20 μL using Taq DNA Polymerase 2× Master Mix RED (Ampliqon, Odense, Denmark). The final MgCl_2_ concentration was adjusted to 1.5 mm. The specific primers (Bioneer Corporation) used for the amplification of full‐length *Pdx1* gene included 5′‐ATATAAGCTTGATATGGAAAGTGAGGAGCAGGAGC‐3′ and 5′‐ATATGGATCCTCACCGGGGTTCCTGCGG‐3′. The primers were designed using primer premier 5.0 software (Premier Biosoft, Palo alto, CA, USA) with restriction sites at the 5′ (HindIII) and 3′ (EcoRI) ends. The PCR was carried out using a thermal cycler (Eppendorf Mastercycler International, Hamburg, Germany). The thermal program given consisted of 35 cycles as follows: initial denaturation at 95 °C for 5 min, denaturation at 94 °C for 1 min, annealing at 58 °C for 1 min, extension at 72 °C for 1 min, and a final extension at 72 °C for 5 min. The purification of the *Pdx1* PCR product from agarose gel was performed using the Gel DNA Recovery Kit (SinaClon BioSciences, Tehran, Iran) as per the manufacturer's recommendations. The purified *Pdx1* PCR product and pcDNA3.1+ vector (ThermoFisher Scientific) were double‐digested with EcoRI and HindIII restriction enzymes (Fermentas, Waltham, MA, USA) at 37 °C for 2 h. The digested fragments were electrophoresed on 1% agarose gel stained by Safe stain (SinaClon BioSciences). The digested fragments were purified using the Gel DNA Recovery Kit (SinaClon BioSciences) based on the manufacturer's instructions. The purified linear vector and insert were subjected to ligation reaction using T4 DNA ligase (Fermentas). The reaction was deactivated through incubation at 65 °C for 15 min. Two microliters of the ligation product was transformed into calcium chloride‐competent *Escherichia coli* Top10F’ cell (Clontech Laboratories, Inc., Takara Holdings, Kyoto, Japan). The transformed cells were selected on lysogeny broth (LB) medium agar plates using ampicillin (100 μg·mL^−1^). Several colonies were assayed by colony PCR using the universal primers T7 and BGH. Positive recombinant clones were cultured overnight at 37 °C. The plasmid was purified using the AccuPrep Nano‐Plus Plasmid Mini Extraction Kit (Bioneer Corporation, Daejeon, South Korea) and sequenced using the BigDye Terminator v3.1 Cycle Sequencing Kit in an ABI 3130 Genetic Analyzer (Applied Biosystems, Waltham, MA, USA).

### Determination of functionality of *Pdx1‐*pCDNA3.1(+) vector

To ensure the efficient overexpression of Pdx1 protein in eukaryotic cells, Chinese hamster ovary (CHO) cells were transfected with *Pdx1‐*pCDNA3.1(+) vector and the expression of *Pdx1* was determined using western blot analysis. CHO cells were cultured in Dulbecco's modified Eagle's medium (DMEM)‐HG medium containing 10% FBS and 1% Pen/Strep. After reaching a confluence of 70%, the cells were transfected with 20 μg of purified *Pdx1*‐pCDNA3.1(+) recombinant vector using electroporation. A gene pulser (Bio‐Rad, Hercules, CA, USA) was used to produce a pulse of 140 V for 15 msec. The CHO cells transfected with pCDNA3.1(+) were used as the control group. The transfected cells were selected using 1.5 mg·mL^−1^ of neomycin. The cells were incubated at 37 °C for 2 weeks. The medium was changed every 3 days. The transfected CHO cells were centrifuged for 5 min at 448 ***g*** washed three times with sterile PBS, and mixed with 100 μL of RIPA buffer (50 mm HCl, 150 mm NaCl, 0.1% Triton X‐100, 0.1% SDS, 1 mm EDTA, 1 mm NaF, and 1 mm PMSF in ddH_2_O). After 30 min of incubation on ice, the samples were centrifuged for 20 min at 11 200 ***g***. The supernatant was collected for further analysis. The total protein concentration was determined using the Bradford method and BSA (1 mg·mL^−1^) as the standard solution. A total of 10 μL of cell lysate was mixed with 10 μL of Laemmli sample buffer containing 7.5% β‐mercaptoethanol and was heated for 10 min at 70 °C. The samples were loaded onto 12% SDS/PAGE gel and transferred into a nitrocellulose membrane (Schleicher & Schuell, Inc., Keene, NH, USA). The membranes were blocked with 5% nonfat milk in PBS. After washing with PBS/Tween, the primary anti‐Pdx1 and GAPDH antibodies (Abcam, Cambridge, MA, USA) were added at the ratio of 1 : 1000. The primary antibody detection was performed using goat anti‐rabbit HRP‐conjugated antibody (Abcam) at a 1 : 1000 ratio. The membranes were incubated with diaminobenzidine (DAB) for 10 min at room temperature and dried at a 37 °C incubator for 15 min. The densitometric quantification of Pdx1 protein in relation to GAPDH as the calibrator was performed using imagej software (National Institutes of Health, Bethesda, MD, USA). The western blot analysis was performed in three independent experiments for each sample.

### Isolation and culture of ADMSCs

The adipose tissue was harvested from the perivesical region and preserved in cold PBS containing 3% Pen/Strep. The adipose tissue was minced under sterile conditions and digested with collagenase type 1 (Alfa Aesar, Ward Hill, MA, USA) for 25 min at 37 °C by shaking. The cell suspension was centrifuged at 161 ***g*** for 8 min, and the supernatant was discarded. The pellet of the cells was resuspended in DMEM containing high glucose and 1% Pen/Strep and 10% FBS and was transferred to 25‐mL culture flasks. The flasks were incubated at 37 °C with 5% CO_2_. Four days after isolation, the medium was changed.

### Characterization of ADMSCs

#### Fluorescence‐based assay

At the third passage, after the number of cells reached 1 × 10^6^ cells·mL^−1^, the cells were washed three times with PBS and harvested using 0.05% trypsin. The cells were washed three times with PBS. Specific antibodies against cell surface clusters of differentiation (CDs; CD34, CD45, CD90, and CD105) were dissolved in 3% BSA/PBS. A total of 1 μg·mL^−1^ of the antibody solution was added to the cells. The samples were incubated in the dark at room temperature for 30 min. The cells were washed three times with PBS and resuspended in 1 mL of ice‐cold PBS containing 10% FBS and 1% sodium azide. The fluorescence activity of the samples was measured using a Galaxy flow cytometer (Dako, Troy, MI, USA). The results were analyzed with flowjo 8.8.7 software (Treestar, Inc, Ashland, OR, USA). Two negative controls, including an isotype control and a stainless control, were provided for each sample.

#### Adipogenic differentiation of ADMSCs

At the third passage, for the adipogenic differentiation of ADMSCs, an adipogenic medium containing 0.5 mm isobutylmethylxanthine (Sigma‐Aldrich, St. Louis, MO, USA), 1 μm dexamethasone (Sigma‐Aldrich), 10 μm insulin (Roche, Basel, Switzerland), 100 μm indomethacin (Sigma‐Aldrich), and 10% FBS in DMEM‐LG (Gibco) was added. The cells were kept in the adipogenic medium for 21 days. The medium was refreshed every 3 days. After completing the differentiation process, the cells were washed three times with PBS. Oil Red O was dissolved in isopropanol and added to the flasks. The flasks were incubated at room temperature for 1 h. The cells were washed with PBS, and the oil droplets were visualized by light microscopy.

#### Osteogenic differentiation of ADMSCs

At the third passage, ADMSCs were differentiated to osteocytes using an osteogenic medium containing 50 μg·mL ascorbic acid (Sigma‐Aldrich), 10 mm β‐glycerophosphate (Sigma‐Aldrich), 10 nm dexamethasone (Sigma‐Aldrich), and 10% FBS in DMEM‐LG. The differentiation medium was changed every 3 days. After 14 days, the cells were washed three times with cold PBS and stained with 40 mm Alizarin Red. The presence of calcium deposits was examined using light microscopy.

### *In vitro* differentiation of ADMSCs to IPCs

**Figure 1:**
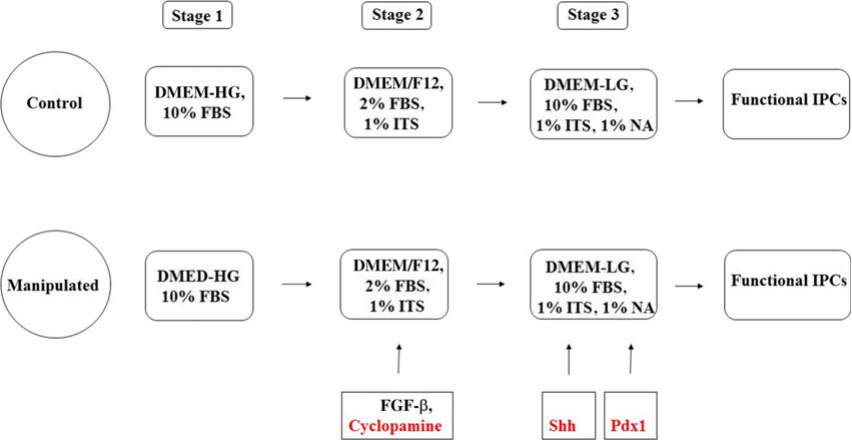
Differentiation protocol of ADMSCs into IPCs. ADMSCs in the control group were differentiated into IPCs using the three‐stage basic protocol. Cells in the manipulated group were treated with 0.25 μm cyclopamine and 64 ng·mL^−1^ FGF‐β at day 3 of differentiation for suppression of Shh pathway and were subsequently treated with recombinant Shh at day 11 of differentiation for reactivation of Shh pathway. Manipulated cells were transfected with *Pdx1‐* pCDNA3.1(+) recombinant plasmid at day 10 of differentiation.

The differentiation protocol used is summarized in Fig. 1. ADMSCs at the third passage were divided into two separate groups: group C (the control group) and group M (the manipulated group). The basic differentiation protocol was performed in the control group. In the manipulated group, the differentiation protocol consisted of the basic protocol plus the sequential inactivation and reactivation of the Shh pathway and *Pdx1* overexpression. The basic protocol consisted of a 7‐day treatment with DMEM/F12, 1% insulin/transferrin/selenium (ITS), 2% FBS, and the change of the medium with a mixture of DMEM‐LG, 1% nicotinic acid (NA), 1% ITS, and 10% FBS for the subsequent 7 days 12, 13. In the manipulated group, Shh was inhibited at day 3 and subsequently reactivated at day 11 of differentiation. The inhibition cells were cultured in a medium containing 64 ng·mL^−1^ FGF‐β (Sigma‐Aldrich) and 0.25 μm cyclopamine (Sigma‐Aldrich). The reactivation of Shh was performed at day 11 through the incubation of the cells in a medium containing 150 ng·mL^−1^ recombinant Shh (Sigma‐Aldrich). The overexpression of *Pdx1* was performed at day 10 of differentiation by the transfection of the cells with *Pdx1‐*pCDNA3.1(+) vector as described in the ‘Determination of functionality of *Pdx1‐*pCDNA3.1(+) vector’ section. The differentiation medium was refreshed every 2 days.

### Expression of pancreatic β‐cell‐related genes

Real‐time PCR was performed to compare the expression of the genes related to the pancreatic β cells (*Pdx1, Nkx2.2. Nkx6.1, MafA, Isl1, Ngn3*, and *insulin*) between the two groups. The analysis was performed using the Ampliqon RealQ Plus Master kit (Ampliqon) and the LightCycler® SYBR Green I Master (Ampliqon). The primer (Bioneer Corporation) characteristics are listed in Table 1. The relative expression of the genes related to the pancreatic β cells was compared using GAPDH as the housekeeping gene. The reactions were prepared in a 25 μL mixture containing 12.5 μL Master Mix kit, 0.5 μL of each primer (200 nm), 3 μL cDNA (100 ng), and 8.5 μL nuclease‐free water. The PCR protocol consisted of a 5‐min denaturation at 95 °C followed by 45 cycles at 95 °C for 15 s and at 60 °C for 30 s. Two separate reactions without cDNA or with RNA were performed in parallel as the controls. Relative quantification was performed according to the comparative 2^−ΔΔCt^ method using lightcycler 96® software. The validation of the assay to check whether the primer had similar amplification efficiencies for the target genes and GAPDH was performed as previously described 14, 15. All the qPCR analyses were performed according to the Minimum Information for Publication of Quantitative Real‐Time PCR Experiments (MIQE) guidelines 16.

**Table 1:** Characteristics of primers that were used for real‐time PCR analysis

Gene name	Sequence (5′→3′)	Length (bp)	Accession number
GAPDH	TG GTATCGTGGAAGGACTC	290	NM_017008.4
	CCTGCTTCACCACCTTCTTG		
Pdx1	GGAGGGTTTGGAAAACCAGT	131	NM_022852.3
	ACAAACATAACCCGAGCACA		
Nkx2.2	AAACCGTCCCAGCGTTAAT	126	NM_001191904.1
	TGCTTTAGAAGACGGCTGAC		
Nkx6.1	ACACACGAGACCCACTTTTT	147	NNM_031737.1
	TTCTGGAACCAGACCTTGAC		
Isl1	GCTTTTCAGCAACTGGTCA	123	NM_017339.3
	AATAGGACTGGCTACCATGC		
Insulin	ATCTTCAGACCTTGGCACTG	141	NM_019129.3
	ATCTTCAGACCTTGGCACTG		
MafA	CTGCTGTCCTACTATGCTCA	137	XM_006241903.2
	TGTATTTCCCCAGGAGTTACAG		
Ngn3	CTATTCTTTTGCGCCGGTAC	128	NM_021700.1
	CTGACGGTCACTTGGCAG		

### Evaluation of IPCs’ functionality *in vitro*

#### Insulin release assay

The IPCs’ ability for insulin secretion was assayed using ELISA. At day 14 of differentiation, the IPCs were washed three times with Krebs‐Ringer bicarbonate buffer (KRB) containing 120 mm Nacl, 5 mm KCl, 2.5 mm CaCl_2_, 10 mm HEPES, 1.1 mm NaHCO_3_, and 0.5% BSA (Sigma‐Aldrich) in sterile deionized water. The samples were incubated with KRB containing 5.5 mm glucose at 37 °C for 30 min. The supernatant was then exchanged with KRB containing 25 mm glucose and 30 mm KCl, and the incubation was performed at 37 °C for 30 min. The supernatant was collected, and the insulin concentration was determined using a rat‐specific insulin ELISA kit (Monobind, Inc, Lake forest, CA, USA) based on the manufacturer's recommended protocol. The insulin concentration was reported in μIU·mL^−1^.

#### DTZ staining

A working solution of 100 ng·mL^−1^ of dithizone (DTZ; Sigma‐Aldrich) was prepared in dimethyl sulfoxide (Sigma‐Aldrich) and filtered through a 0.2‐μm filter. At the last stage of differentiation, 3 mL of working solution was added to each 25‐cm^2^ flask and incubated at 37 °C for 30 min. The cells were washed three times with PBS, and crimson red‐stained clusters were observed using a phase contrast microscope (Olympus IX71, Tokyo, Japan).

### Transplantation of IPCs

Normal male Sprague‐Dawley rats (*n* = 15) aged 8 weeks and weighing 180–200 g were used. A total of 50 mg·kg^−1^ of streptozotocin (STZ; Sigma‐Aldrich) was dissolved in citrate buffer and injected intraperitoneally to the rats to induce experimental diabetes mellitus. One week after the injection, the rats with basal blood glucose above 500 mg·mL^−1^ were taken to be diabetic. The rats were divided into three groups. One group (*n* = 5) received undifferentiated ADMSCs, another control group (*n* = 5) was injected with unmanipulated IPCs, and the remaining rats (*n* = 5) received manipulated IPCs. The differentiated IPCs (manipulated and unmanipulated) were trypsinized and washed three times with PBS at day 14 of differentiation. Then, 1 × 10^6^ of the isolated cells were suspended in 200 μL of DMEM‐HG. A mixture of 100 mg·kg^−1^ ketamine and 10 mg·kg^−1^ xylazine was used to anesthetize the rats. The cells were injected to the tail vein of the rats. Fasting blood glucose concentrations were measured once a week using a glucometer (EasyGluco, Anyang, South Korea). At the end of the sixth week, the rats received a 25 mm glucose solution. After 10 min, the rats were anesthetized with a mixture of 100 mg·kg^−1^ ketamine and 10 mg·kg^−1^ xylazine. Then, 2 mL of blood was taken from them. The rats’ blood serums were isolated by centrifugation at 2000 r.p.m. for 5 min, and their insulin concentrations were determined using ELISA.

### Statistical analyses

Data analyses were carried out using the spss 18.0 software package (SPSS Inc., Chicago, IL, USA). All analyses were carried out in triplicate. One‐way ANOVA followed by Tukey's post hoc analyses was used to test differences between various means including the expression level of different genes and insulin concentration. All experimental data were presented as mean ± SEM. The level of significance for all tests was set at *P* < 0.05.

## Results

### Characteristics of *Pdx1‐*pCDNA3.1(+) vector

**Figure 2:**
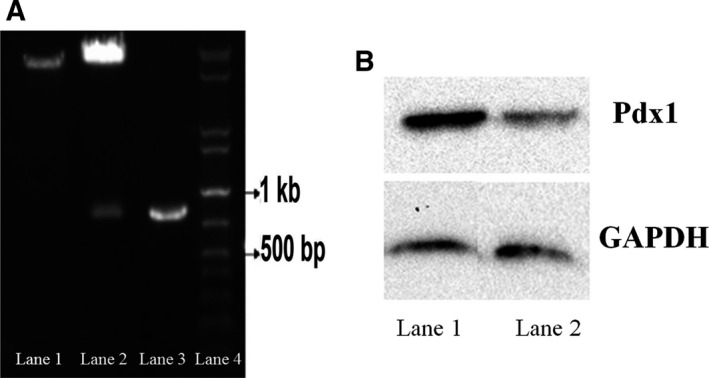
(A) Characterization of *Pdx1‐* pCDNA3.1(+) vector. Lane 1: the recombinant plasmid before digestion. Lane 2: the 850‐bp *Pdx1* gene separated from the recombinant *Pdx1‐* pCDNA3.1(+) digested using EcoRI and HindIII enzymes. Lane 3: 900‐bp PCR product of recombinant *Pdx1‐* pCDNA3.1(+) vector. Lane 4: 1‐kb DNA ladder. (B) Western blot analysis results. The CHO cells transfected with *Pdx1‐* pCDNA3.1(+) (lane 1) showed a significantly higher expression of Pdx1 compared with the CHO cells transfected with pCDNA3.1(+) alone (Lane 2).

As illustrated in lane 2 in Fig. 2A, the *Pdx1* gene is broken from both ends by restriction enzymes and the 850‐bp band is separated from the vector, which suggests the existence of *Pdx1* gene in the pCDNA3.1(+) vector. After transforming the recombinant pCDNA3.1(+) plasmid containing the *Pdx1* sequence to *E. coli*, direct colony PCR was used to ensure an accurate transformation. As shown in lane 3 in Fig. 2A, a 900‐bp band was seen on 1% electrophoresis gel corresponding to the 850‐bp *Pdx1* gene and 50‐bp flanking regions of the plasmid. This finding confirms the accuracy of the recombinant plasmid transformation in the bacteria. The sequencing of the recombinant plasmids was also performed with universal primers to confirm the accuracy of the *Pdx1* sequence after amplification and cloning. The sequence obtained was translated using ExPASy Translate tool (https://web.expasy.org) and then analyzed using the online tools nBLAST and pBLAST. Based on this finding, the cloned *Pdx1* gene sequence had a 100% homology to the *Pdx1* sequences submitted to the GenBank (GenBank accession number: NM_022852.3). The ability of the CHO cells transfected with the *Pdx1*‐pCDNA3.1(+) construct for *Pdx1* expression was confirmed through western blot analysis. After applying specific Pdx1 antibody, a single band at ~ 40 KD was detected. The CHO cells transfected with *Pdx1‐*pCDNA3.1(+) expressed higher levels of *Pdx1* compared with the pCDNA3.1(+)‐transfected cells. *Pdx1* overexpression in CHO cells transfected with the *Pdx1*‐pCDNA3.1(+) construct was thus confirmed (Fig. 2B).

### Characterization of ADMSCs

**Figure 3:**
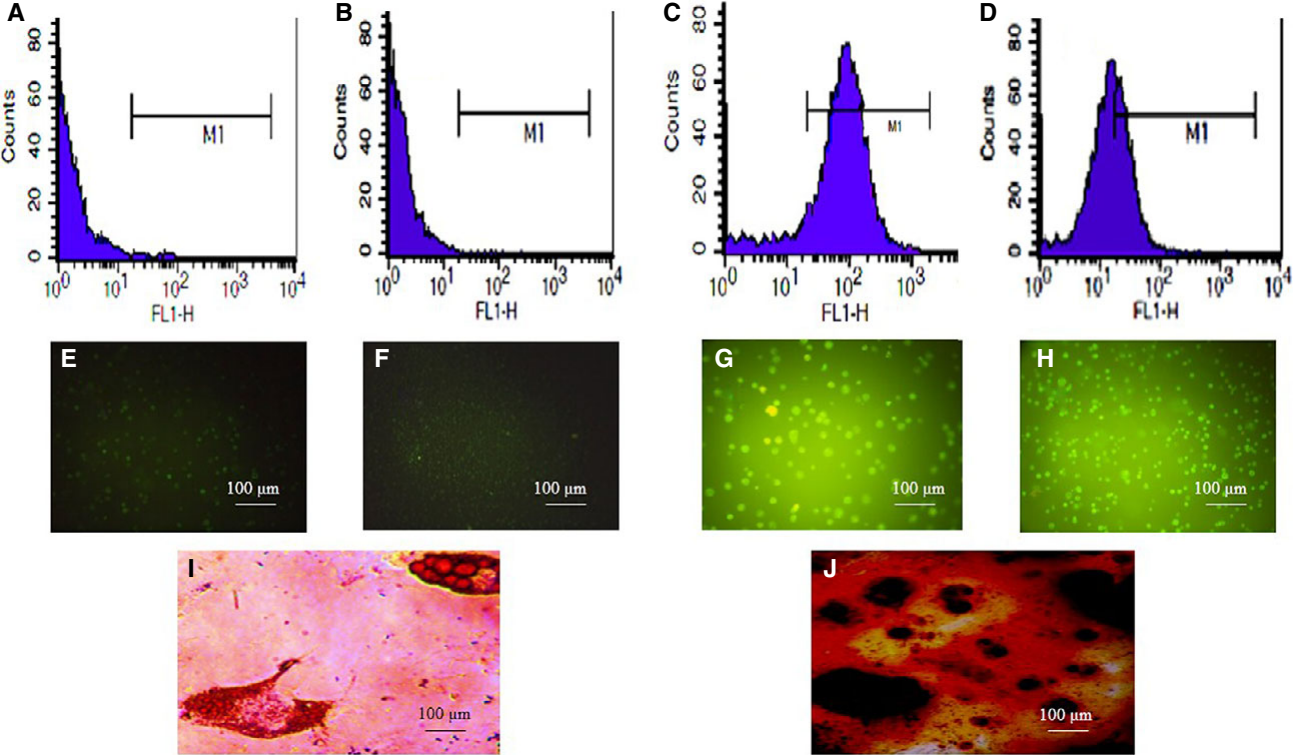
Characterization of ADMSCs. Flow cytometry analysis of CDs markers in isolated ADMSCs showed that undifferentiated ADMSCs were positive for MSC‐specific surface marker proteins including CD90 (C,G) and CD105 (D,H) and negative for hematopoietic and leukocyte common antigens including CD34 (A,E) and CD45 (B,F). The presence of Oil Red O‐stained oil droplets confirmed the adipogenic differentiation potential of ADMSCs (I). Osteogenic differentiation capacity of ADMSCs was confirmed by formation of Alizarin Red‐stained calcium phosphate deposits (J). Phase contrast magnification, 100×.

The results of the fluorescence absorbance assay using flow cytometry and fluorescence microscopy revealed that ADMSCs express specific surface cell markers of multipotent MSCs (CD90; Fig. 3C,G) and CD105 (Fig. 3D,H). The cells lacked the hematopoietic marker CD34 (Fig. 3A,E) and leukocyte common antigen (CD45; Fig. 3B,F). The use of an adipogenic differentiation medium successfully induced the differentiation of ADMSCs to adipocytes. The morphology of ADMSCs gradually changed to a round shape. Consistent with the progress of differentiation, some accumulations of cells were visualized. The Oil Red O staining of the cells at day 21 revealed the accumulation of cytoplasmic fat droplets (Fig. 3I). The ADMSCs’ potency for osteogenic differentiation was verified using an osteogenic medium. After 14 days of induction, the morphology of the cells changed to a fibroblast‐like appearance. The staining of the cells differentiated with Alizarin Red showed the formation of calcium phosphate deposits (Fig. 3J).

### Evaluation of differentiation stages

**Figure 4:**
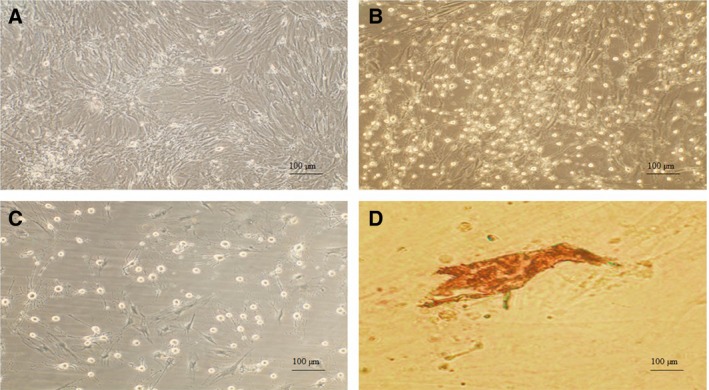
Morphological characteristics of ADMSCs during the differentiation into IPCs. Spindle‐like ADMSCs were shortened and aggregated at day 3 of differentiation (A). Cells presented an epithelial‐like morphology at day 7 of differentiation (B). Cells exhibited an epithelial cell morphology at day 14 of differentiation (C). IPCs stained as crimson red by DTZ at late stages of differentiation (D). Phase contrast magnification, 100×.

**Figure 5:**
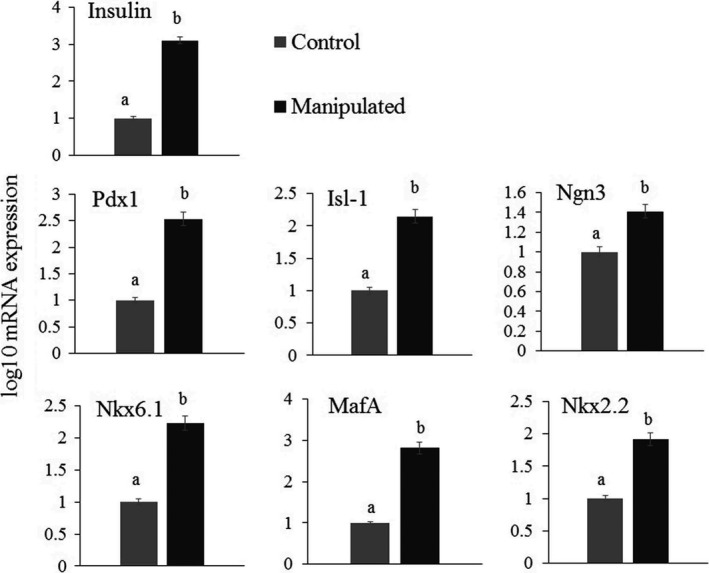
Expression of pancreas‐related genes after Shh pathway manipulation and *Pdx1* overexpression: Manipulation of Shh caused an increase in *Pdx1, MafA, Nkx2.2, Nkx6.1, Ngn3, Isl1*, and *insulin* expression compared with the control group (*P* < 0.05). GAPDH was used as the calibrator for real‐time PCR analysis. Data are expressed as mean ± SE. Statistical significance difference at *P* < 0.05 is represented by different letters.

During the differentiation process, the size of the cells moderately decreased. The cells exhibited a decreased proliferation tendency. At day 3 of differentiation, some accumulations of spindle‐like cells could be distinguished (Fig. 4A). At day 7 of differentiation, the cells showed a round appearance and some aggregations of cells were visualized (Fig. 4B). At day 14 of differentiation, the cells resembled epithelial cells (Fig. 4C). The overexpression of *Pdx1* plus Shh manipulation proved beneficial to the expression of critical genes involved in mature pancreatic β cells. Group M presented an important elevation in the expression of *Pdx1*,* MafA, Ngn3, Isl1, Nkx2.2*, and *Nkx6.1* mRNA compared with group C. Group M showed a significantly higher expression of insulin mRNA compared with group C (Fig. 5).

### Evaluation of IPCs’ functionality *in vitro*

**Figure 6:**
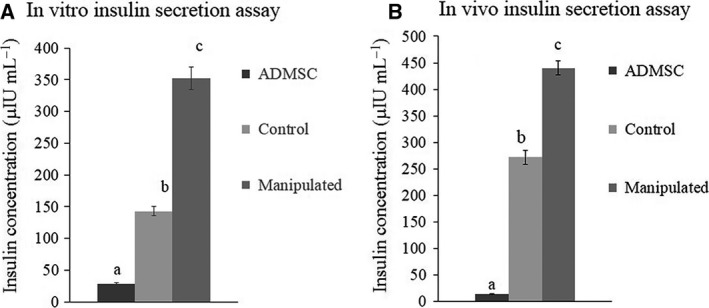
The results of insulin secretion assay. Undifferentiated ADMSCs showed no obvious ability for insulin secretion in response to glucose (A,B). The manipulated group showed a higher expression and secretion of insulin in comparison with the control group *in vitro* (*P* < 0.05) (A). The rats that received manipulated IPCs secreted significantly higher amounts of insulin in response to glucose when compared with the rats that received control IPCs (*P* < 0.05) (B). Statistical significance difference at *P* < 0.05 is represented by different letters.

The fully differentiated cells displayed secretory insulin vesicles stained as crimson red after the DTZ treatment (Fig. 4D). The IPCs obtained from the control or manipulated cells exhibited an insulin secretion ability in response to glucose. Meanwhile, the undifferentiated ADMSCs were unable to secrete insulin. Group M secreted significantly higher amounts of insulin compared with group C (Fig. 6A).

### Evaluation of IPCs’ functionality *in vivo*

**Figure 7:**
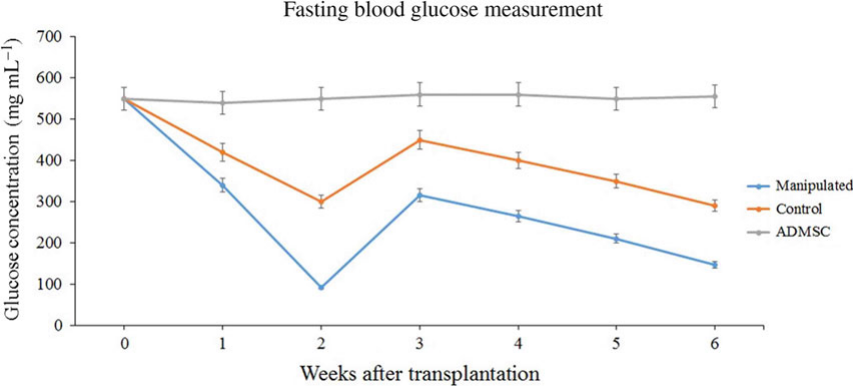
Fasting blood glucose in rats treated with different IPCs. After transplantation of manipulated IPCs to STZ‐diabetic rats (*n* = 5), a sharp reduction in the mean blood glucose concentration (92 ± 1.2 mg·mL^−1^) within 2 weeks was noticed. At the end of the third week, the average amount of blood glucose concentration raised to 315 ± 1.9 mg·dL^−1^. Thereafter, blood glucose concentration decreased gradually. At the sixth week after transplantation, the average amount of glucose concentration reached 147 ± 1.1 mg·mL^−1^. The rats that received positive control IPCs (*n* = 5) followed the same pattern of blood glucose concentration changes. However, they showed a more severe hyperglycemic condition. The diabetic rats that received undifferentiated ADMSCs showed no detectable change in the mean blood glucose concentration.

The transplantation of the manipulated IPCs to STZ‐diabetic rats resulted in a sharp reduction in the mean blood glucose concentration (92 ± 1.2 mg·mL^−1^) within 2 weeks. At the end of the third week, the average amount of blood glucose was raised to 315 ± 1.9 mg·dL^−1^. From then on, blood glucose decreased gradually. At the sixth week after the transplantation, the average glucose concentration reached 147 ± 1.1 mg·mL^−1^. The rats that received positive control IPCs showed the same pattern of changes in blood glucose. Nonetheless, they showed a more severe hyperglycemic condition. The diabetic rats that received undifferentiated ADMSCs showed no detectable changes in their blood glucose concentration (Fig. 7). The measurement of insulin in the blood serum of the experimental rats in response to glucose administration showed significantly higher insulin concentrations in group C and group M compared to undifferentiated ADMSCs. Group M secreted significantly higher amounts of insulin in response to glucose compared with group C (Fig. 6B).

## Discussion

The transplantation of artificial IPCs is a promising treatment for T1DM 17, 18. Nevertheless, the construction of suitable IPCs for clinical use requires further adjustments in differentiation protocols 18. Gene therapy has a great potential in this area 19. Studies suggest the critical role of the Shh pathway as a mediator of the differentiation of embryonic gut endoderm into pancreatic β cells 19. Shh expression is reportedly blocked during pancreatic bud endoderm formation 20. Shh plays an important role in the regulation of proliferation and maintenance of mature pancreatic β cells 10, 21. Recent studies have revealed the beneficial effects of the sequential inactivation and reactivation of Shh on the differentiation of MSCs into IPCs 10, 21. In one study, Thomas *et al*. 10, 11 showed that Shh induces insulin expression and secretion through the mature pancreatic β cells and concluded that Shh affects the *Pdx1* promoter and adjusts *Pdx1* expression, while *Pdx1* in turn plays an important role in the regulation of insulin gene expression. Previous studies have also shown the key role of *Pdx1* in the differentiation of different stem cells into IPCs, the activation of insulin transcription, and the maintenance of IPCs 22. The present study proposes a novel protocol that uses combined *Pdx1* overexpression and Shh manipulation to optimize the differentiation of ADMSCs toward IPCs. The isolated ADMSCs exhibited general fibroblast‐like phenotypes of MSCs. The adherent ADMSCs expanded rapidly and expressed stem cell markers including CD90 and CD105, while they were negative for CD34 and CD45. The multipotency of ADMSCs was confirmed by their successful differentiation into osteocytes and adipocytes. The main differentiation protocol resulted in the production of functional IPCs. The differentiated cells obtained exhibited an endocrine pancreatic cell morphology and insulin secretion capacity in response to glucose. A study by Raikwar *et al*. 23 on the differentiation of *Pdx1*‐engineered embryonic stem cells into IPCs similarly yielded an elevation of *Ngn3, Isl1*, and insulin expression both *in vitro* and *in vivo*. By contrast, the study by Kubo *et al*. 24 on the overexpression of both *Pdx1* and *Ngn3* during the differentiation of embryonic mice cells into IPCs resulted in the production of immature IPCs. The study by Cao *et al*. showed the effect of *Pdx1* overexpression on IPCs’ functionality. The IPCs obtained in the study by Cao *et al*. reversed the hyperglycemic condition but were unable to express the late‐stage genes related to the pancreatic β‐cell development. They concluded that the exact differentiation of hepatic cells into functional IPCs requires additional external factors 25. Thomas *et al*. showed that *Pdx1* has a hedgehog‐responsive element on its promoter. Hedgehog suppression therefore downregulates *Pdx1* and ultimately decreases insulin expression 11. In a previous study by the present researchers, the manipulation of the Shh pathway led to a significant overexpression of *Pdx1, MafA, Ngn3, Isl1, Nkx2.2, Nkx6.1*, and *insulin* mRNA compared to the control group 13. The differentiated cells obtained exhibited a remarkable increase in insulin synthesis and secretion. These results revealed the usefulness of Shh pathway manipulation in improving IPC maturity 11, 20, 26, 27, 28. This study was therefore conducted to examine the effect of *Pdx1* overexpression concurrent with Shh manipulation on differentiation outcomes. The IPCs obtained expressed significantly higher amounts of *Pdx1, MafA, Ngn3, Isl1, Nkx2.2, Nkx6.1*, and *insulin* compared to the control group. The differentiated cells obtained secreted remarkably higher amounts of insulin than the cells in the control group. This finding demonstrates the effectiveness of *Pdx1* overexpression concurrent with Shh manipulation in the production of functional IPCs *in vitro*. The manipulated IPCs obtained in the present study were capable of reducing blood glucose levels to near euglycemia. The potency of the manipulated cells for the secretion of insulin showed a significant increase compared to the control IPCs *in vivo*. In line with the present findings, Thomas *et al*. 11 reported the enhancing effect of Shh on *Pdx1* promoter activity and *Pdx1* and insulin expression at the late stages of pancreatic development. Hebrok *et al*. 29 proved the critical role of Shh in adjusting pancreatic development in a dose‐dependent way. By contrast, the study by Hori *et al*. on the differentiation of human neural progenitor cells into IPCs showed no detectable expression of Shh during the four stages of differentiation toward IPCs. In their study, 300 ng·mL^−1^ of Shh protein administered at the final stage of differentiation resulted in the elimination of *Pdx1, FoxA3*, and *insulin* expression 30. The disparity of findings might be due to the differences in cell sources and Shh concentration.

## Conclusion

The present study reveals successful functional IPC generation through the described differentiation protocol. Shh manipulation and *Pdx1* overexpression induced the generation of functional IPCs. The concurrent use of *Pdx1* overexpression and Shh manipulation significantly increased the functionality of the generated artificial IPCs. Further studies are needed to clarify the molecular mechanisms underlying the concurrent use of *Pdx1* and Shh in the generation of mature functional IPCs.

## Author contribution

DD, MHT, EMP, and MRT designed the study. DD, MHT, and MRT performed the study, researched the data, analyzed the results, wrote the manuscript, and revised the article critically for important intellectual content. EAB, AAG, MAG, and MO analyzed the data and drafted and revised the manuscript. All authors gave final approval of the version to be published. DD is the guarantor of this work and, as such, had full access to all the data in the study and takes responsibility for the integrity of the data and the accuracy of the data analysis.
